# Astrocyte-elevated gene-1 confers resistance to pemetrexed in non-small cell lung cancer by upregulating thymidylate synthase expression

**DOI:** 10.18632/oncotarget.18717

**Published:** 2017-06-28

**Authors:** Chung-Yu Chen, Ying-Yin Chen, Jeremy J.W. Chen, Kuan-Yu Chen, Chao-Chi Ho, Jin-Yuan Shih, Yih-Leong Chang, Chong-Jen Yu, Pan-Chyr Yang

**Affiliations:** ^1^ Department of Internal Medicine, National Taiwan University Hospital Yunlin Branch, Douliu, Taiwan; ^2^ Division of Pulmonary and Critical Care Medicine, Department of Internal Medicine, National Taiwan University Hospital, College of Medicine, National Taiwan University, Taipei, Taiwan; ^3^ Department of Pathology and Graduate Institute of Pathology, College of Medicine, National Taiwan University, Taipei, Taiwan; ^4^ Institute of Biomedical Science, College of Life Sciences, National Chung Hsing University, Taichung, Taiwan

**Keywords:** lung cancer, pemetrexed, thymidylate synthase, astrocyte elevated gene-1, chemoresistance

## Abstract

Previous studies have suggested that astrocyte-elevated gene-1 (AEG-1) contributes to the mechanisms of resistance to various chemotherapeutics. In this study, we investigated whether AEG-1 expression level correlated with that of thymidylate synthase (TS), as higher TS expression is known to be associated with the resistance to pemetrexed chemotherapy in patients with advanced lung adenocarcinoma. Using pemetrexed-resistant lung adenocarcinoma PC-9 cell line, we demonstrated that transfection of AEG-1 siRNA lowered TS expression and decreased pemetrexed IC_50_ value. In contrast, overexpression of AEG-1 was associated with increased expression of TS and higher pemetrexed IC_50_ value. Immunohistochemical staining of clinical biopsy samples showed that patients with lower AEG-1 expression had longer overall survival time. Moreover, analysis of repeated biopsy samples revealed that an increase in the TS level from baseline to disease progression was significantly associated with the elevation of AEG-1 expression. In conclusion, our data demonstrated that TS expression might be regulated by AEG-1 and that increased expression of these proteins contributes to lung cancer disease progression and may be associated with the development of resistance to pemetrexed.

## INTRODUCTION

Lung cancer is the leading cause of cancer deaths worldwide [1, 2]. Non-small cell lung cancer (NSCLC) accounts for 85% of all lung cancer cases, and more than 40% of patients with NSCLC have advanced disease (stage IIIB or IV) and need systemic chemotherapy at the time of diagnosis [3–5]. Pemetrexed is active against non-squamous NSCLC and is widely used in platinum-based doublet as either first-line chemotherapy, maintenance therapy, or further-line chemotherapy [6–9]. Pemetrexed is a multi-targeting anti-metabolite that inhibits enzymes involved in folate metabolism and DNA synthesis, including thymidylate synthase (TS), dihydrofolate reductase, glycinamide ribonucleotide formyltransferase, and aminoimidazole carboxamide ribonucleotide formyltransferase [10], although TS has been considered as the main target. In NSCLC, median level of TS expression is lower in adenocarcinoma than in squamous cell carcinoma [11]. Furthermore, TS levels inversely correlated with treatment efficacy and survival of NSCLC patients treated with pemetrexed [12–19]. High baseline TS expression levels confer resistance to pemetrexed, and higher TS expression also appears to be associated with acquired resistance to pemetrexed treatment [20, 21]. However, the mechanism of TS expression induction in drug resistance to pemetrexed is not fully understood.

Expression of astrocyte-elevated gene-1 (AEG-1), a novel oncoprotein, is elevated in several cancers. AEG-1 plays a vital role in tumor cell growth, invasion, angiogenesis, and progression to metastasis [22, 23]. High expression level of AEG-1 likely promotes carcinogenesis and leads to a poor clinical prognosis of NSCLC [24, 25]. Recent findings have suggested that AEG-1 contributes to a broad-spectrum resistance to various chemotherapeutics [26]. For example, 5-fluorouracil (5-FU) inhibits TS, whereas AEG-1 induces resistance to 5-FU by increasing the expression of TS [27]. Activation of AEG-1 and associated signaling pathways may therefore cause drug resistance in cancer treatment [28, 29].

However, clinical significance and biological role of AEG-1 in NSCLC remain unclear. In fact, the mechanism of TS expression regulation by AEG-1 and its association with the development of resistance to chemotherapy with pemetrexed has not yet been elucidated. We thus investigated the expression of AEG-1 in NSCLC to determine whether it correlates with changes in TS expression. We explored a possible molecular mechanism through which AEG-1 is involved in the regulation of TS expression in NSCLC and established that AEG-1 confers resistance to pemetrexed by inducing TS expression.

## RESULTS

### Expression of AEG-1 positively correlated with TS expression and negatively correlated with sensitivity of NSCLC cell lines to pemetrexed

**Figure 1:**
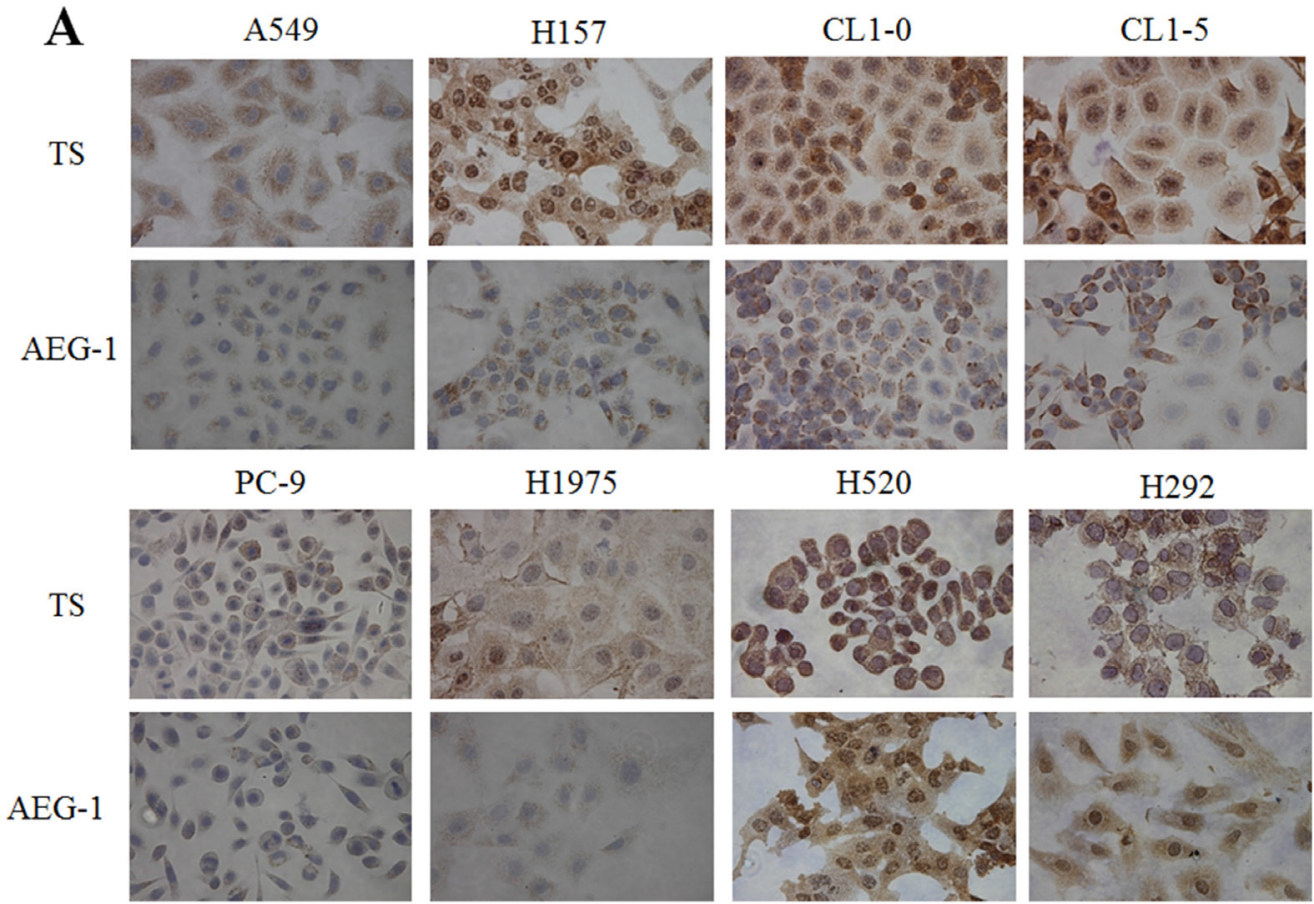

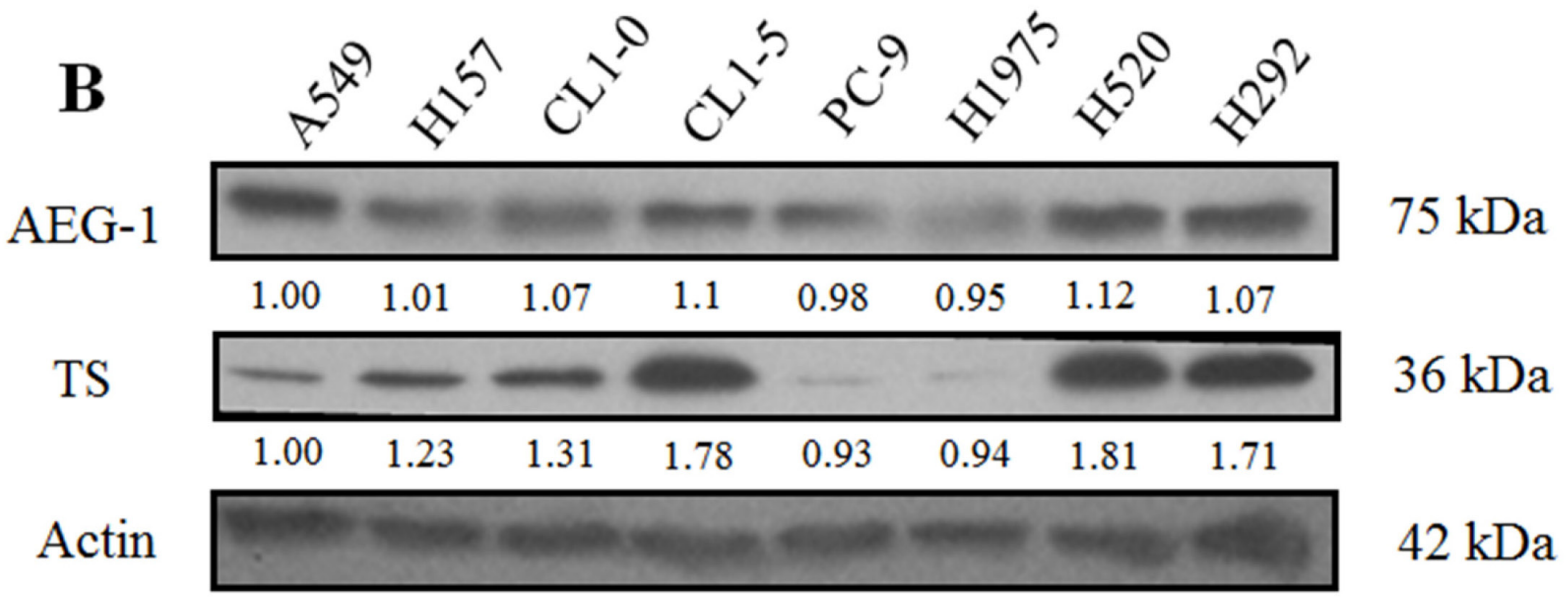

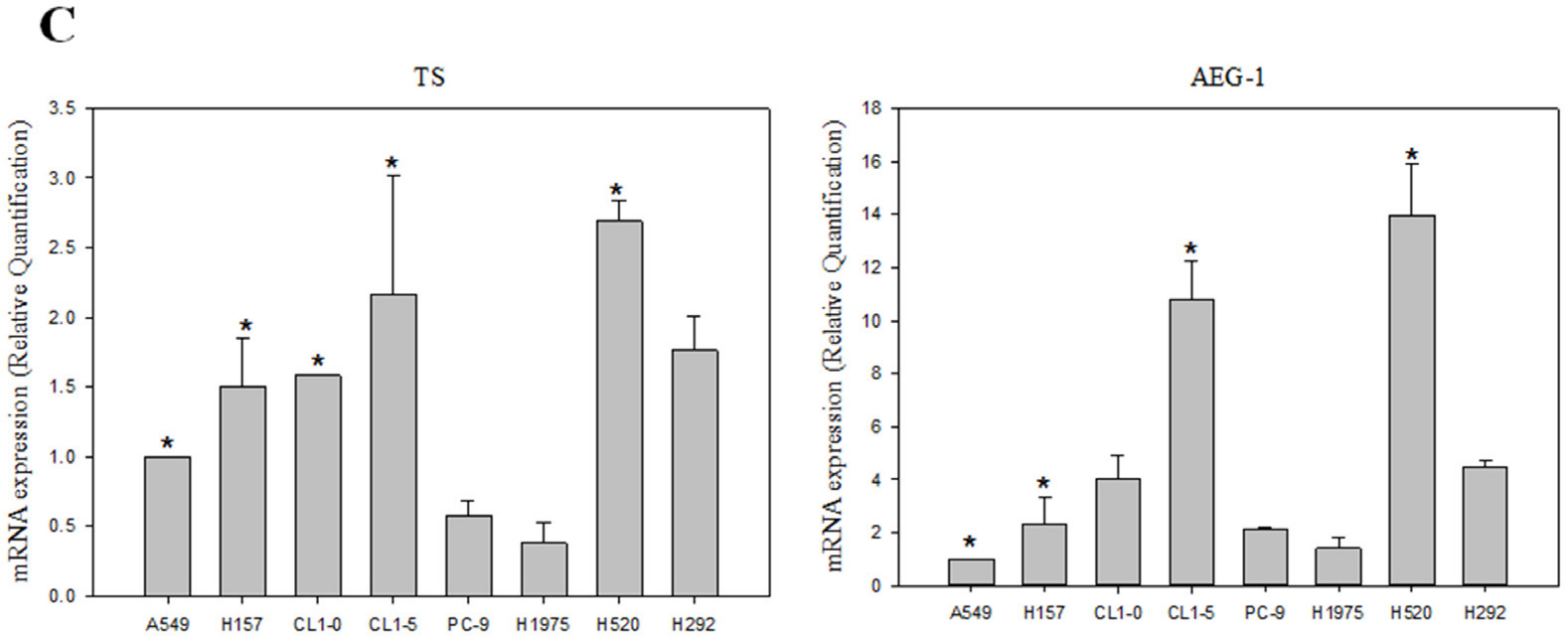

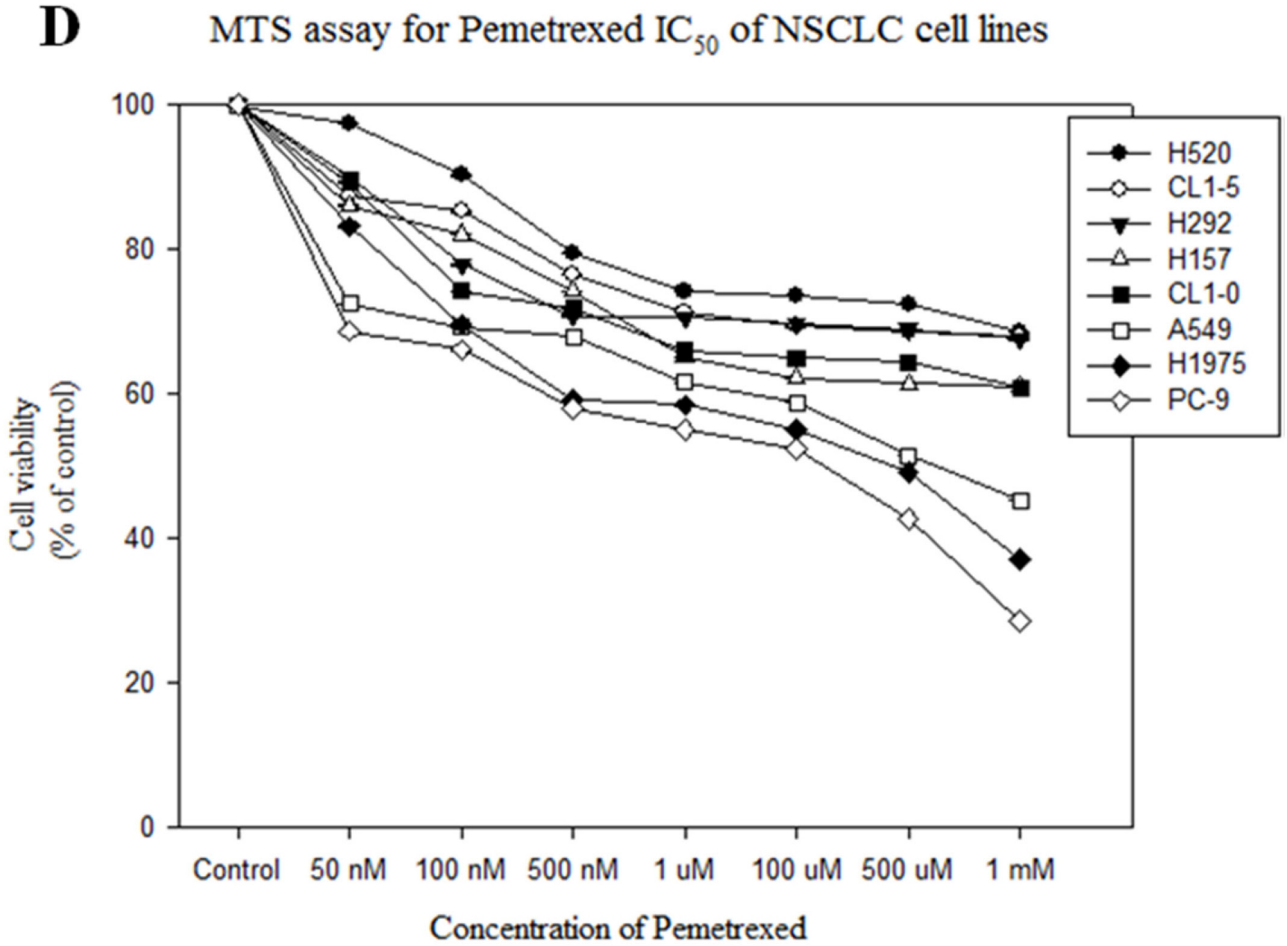

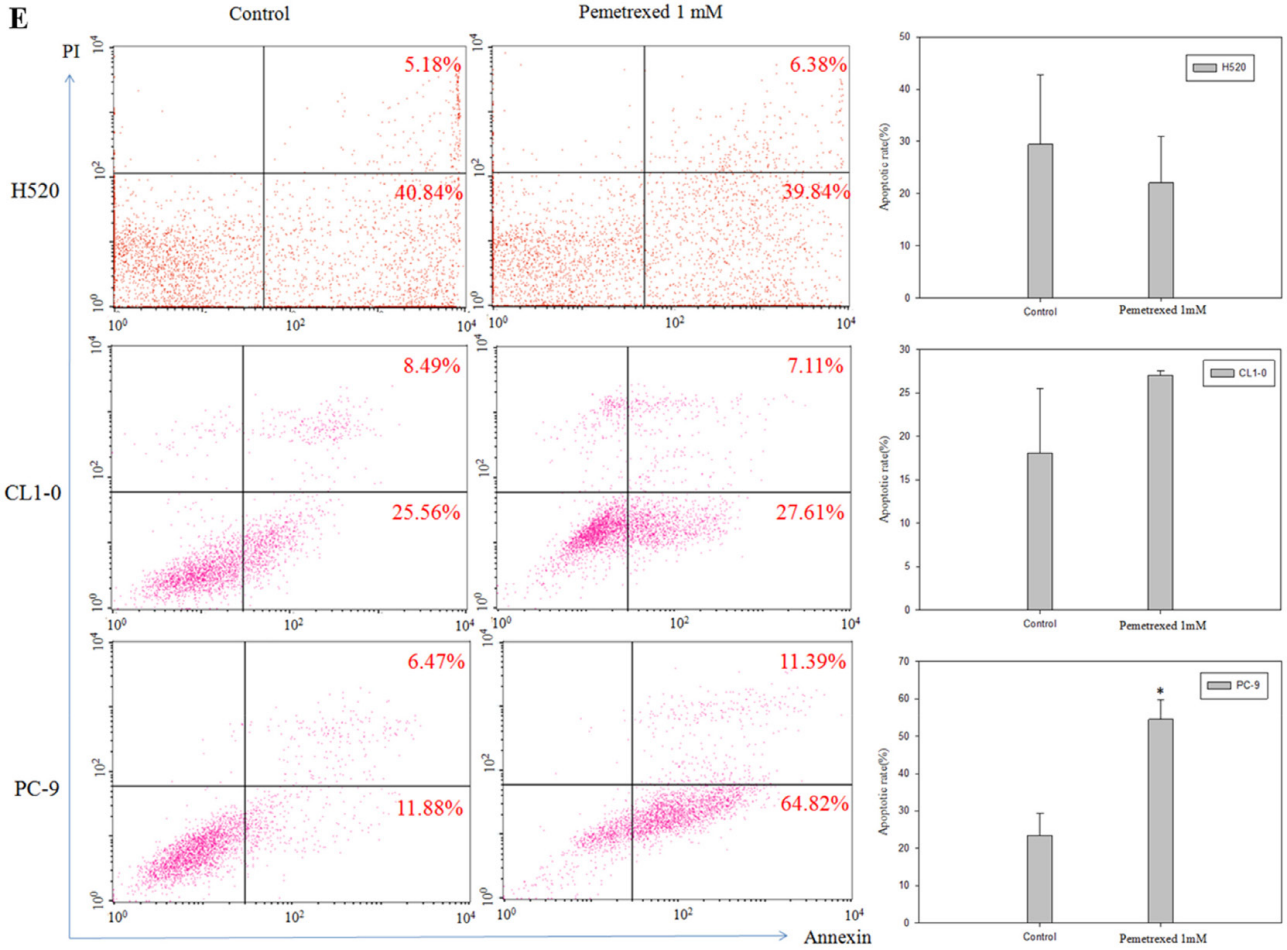
Expression of astrocyte-elevated gene-1 and thymidylate synthase in several non-small cell lung cancer cell lines **(A)** Immunohistochemical staining revealed upregulation of astrocyte-elevated gene-1 (AEG-1) and thymidylate synthase (TS) protein expression in the primary non-small cell lung cancer (NSCLC) cell lines H157, CL1-0, CL1-5, H520, and H292. **(B)** Expression of AEG-1 and TS proteins in NSCLC cell lines was analyzed with western blotting. **(C)** AEG-1 and TS gene expression levels were determined by qPCR. *: compare with PC-9, *p* < 0.05. **(D)** Pemetrexed concentration-cell viability relationship was determined in eight NSCLC cell-lines. Lower pemetrexed IC_50_ values were noted in NSCLC cell lines expressing lower TS levels. **(E)** Flow cytometry dot plots and quantitative analysis showed apoptosis rates in three representative cell lines (H520, CL1-0, and PC-9) with different TS expression levels. Measurements from control cells and cells treated with pemetrexed are shown.*p < 0.05.

We investigated a possible correlation between TS and AEG-1 expression levels in eight NSCLC cell lines. Higher levels of TS and AEG-1 protein and mRNA, revealed by immunohistochemical staining of fixed cells (Figure 1A), western blotting of cell extracts (Figure 1B), and real time - polymerase chain reaction (RT-PCR) (Figure 1C), respectively, were noted in H157, CL1-0, CL1-5, H520, and H292 cell lines. In contrast, A549, PC-9, and H1975 cell lines had lower levels of TS and AEG-1 expression. TS expression levels positively correlated with those of AEG-1 in lung cancer cell lines. Furthermore, pemetrexed IC_50_ values were lower in lung cancer cell lines with lower TS gene expression (Figure 1D). Apoptosis rates were also increased by pemetrexed treatment in lung cancer cell lines with lower TS expression levels (Figure 1E). These results revealed a significant positive correlation between TS and AEG-1 gene expression levels in NSCLC cell lines. Moreover, differences in TS expression levels paralleled differential sensitivity of cell lines to pemetrexed treatment.

### Knockdown of AEG-1 in NSCLC PC-9 cell line reverted pemetrexed resistance *in vitro*

**Figure 2:**
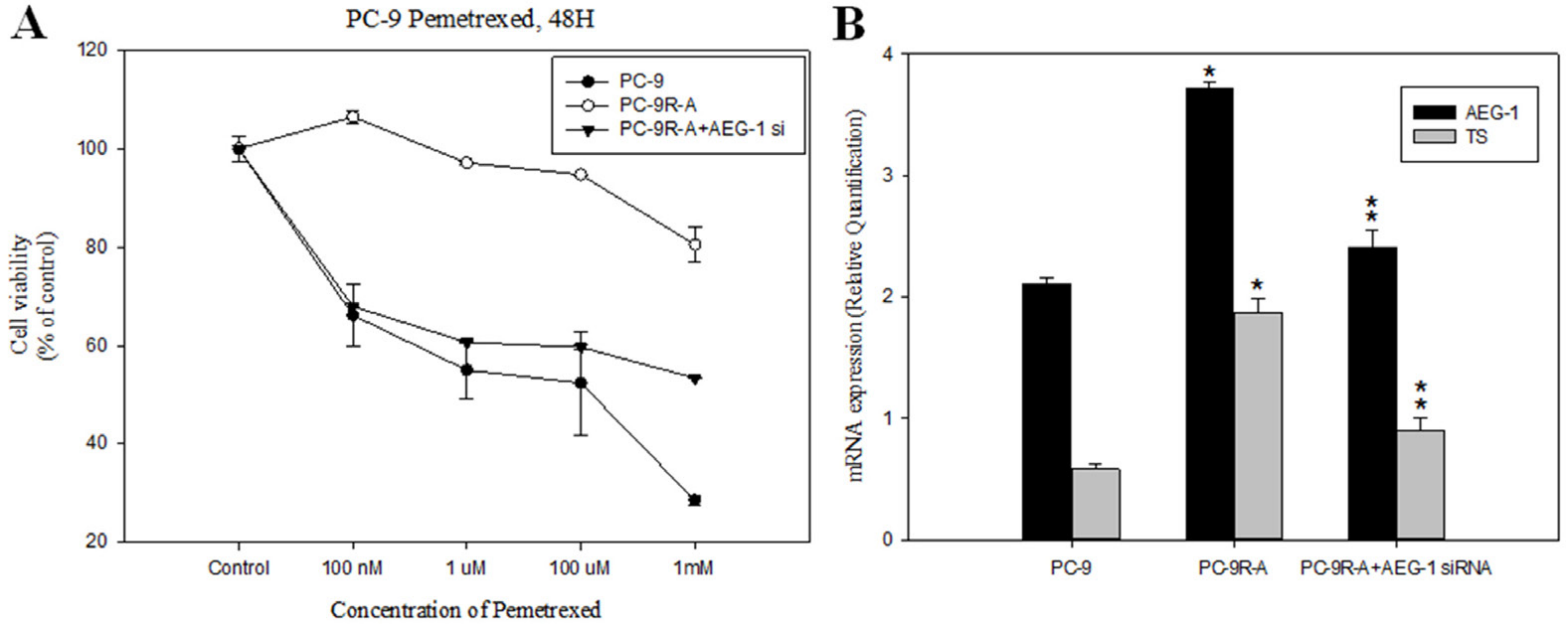

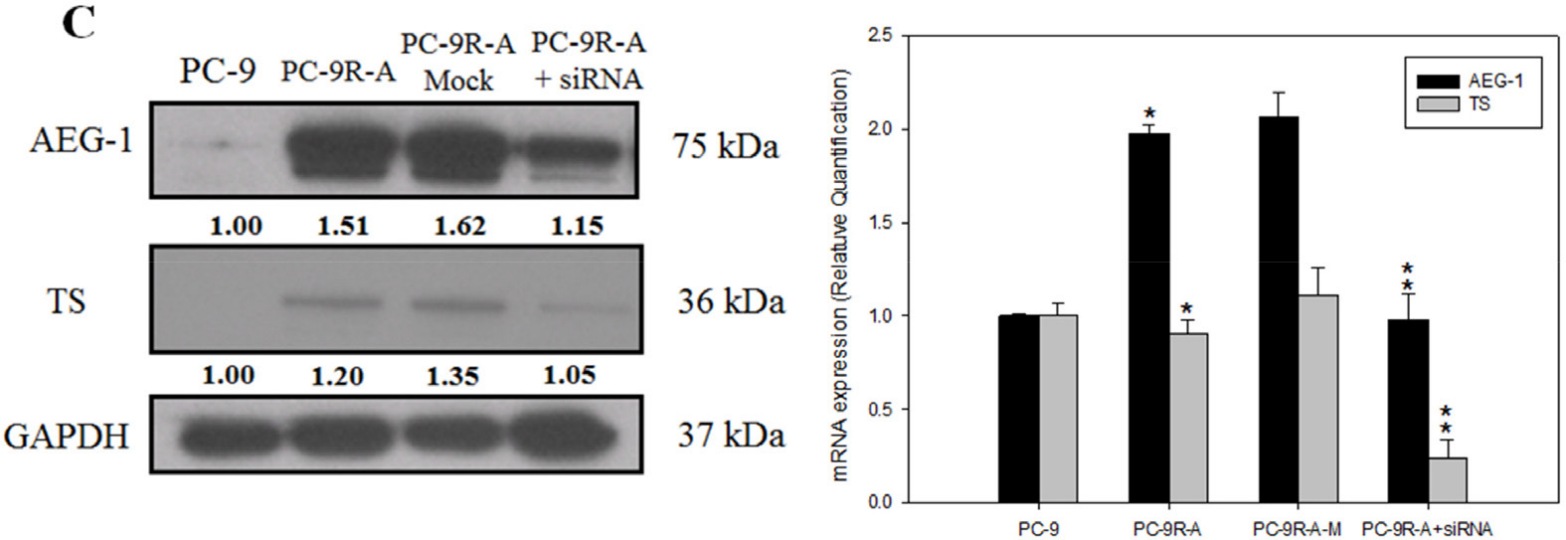

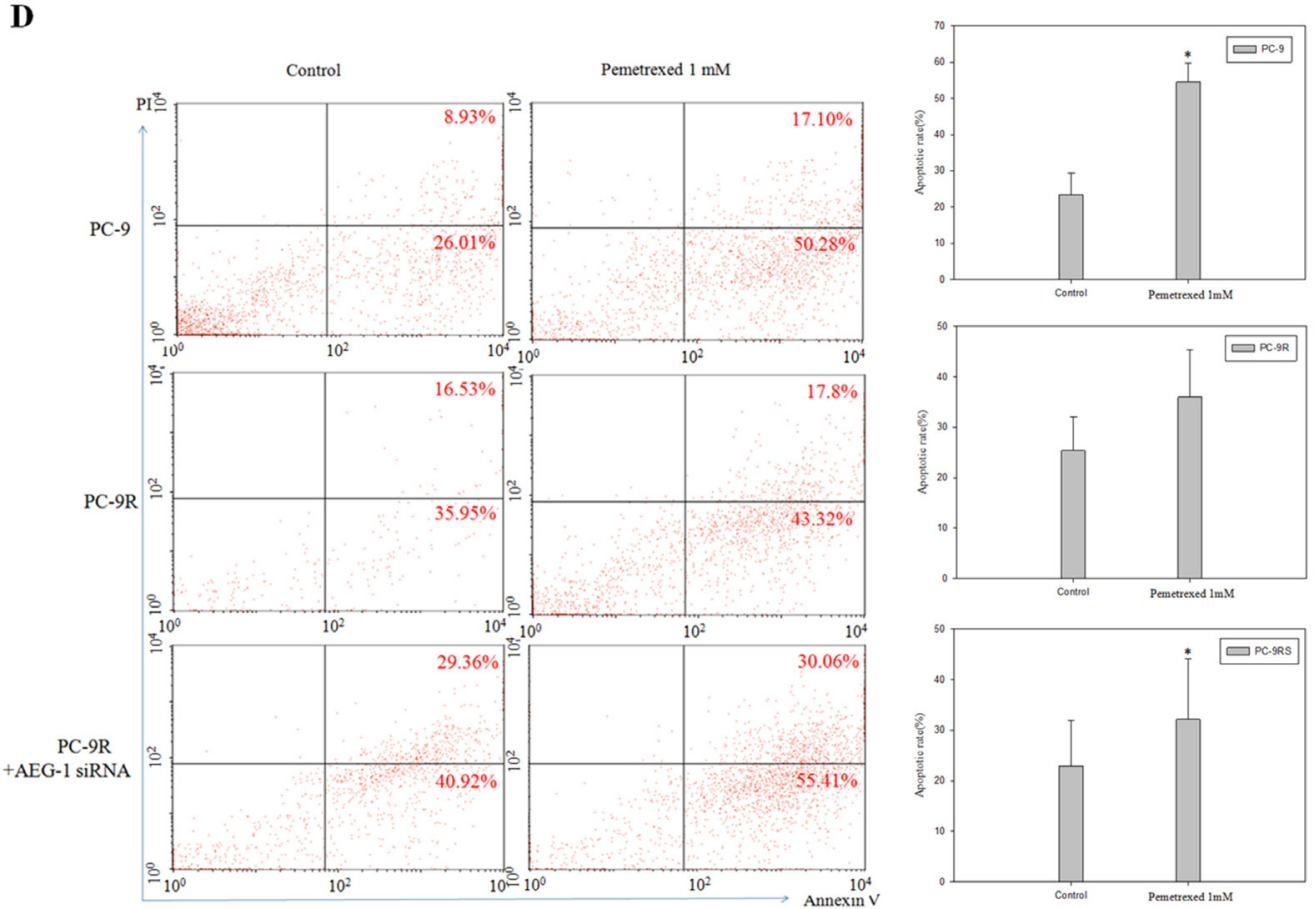
Knockdown of astrocyte-elevated gene-1 in a pemetrexed-resistant non-small cell lung cancer cell line conferred higher sensitivity to pemetrexed **(A)** MTT assay demonstrated the restoration of sensitivity to pemetrexed in a pemetrexed-resistant cell line (PC-9R-A) after siRNA-mediated astrocyte-elevated gene-1 (AEG-1) knockdown (PC-9R-A+AEG-1 siRNA transfection). **(B)** Upregulation of relative AEG-1 and thymidylate synthase (TS) mRNA levels in a pemetrexed-resistant cell line (PC-9R-A) and their sensitivity to siRNA-mediated AEG-1 knockdown (PC-9R-A+AEG-1 siRNA transfection) was revealed by RT-PCR. *: compare with PC-9, *p* < 0.05; **: compare with PC-9R-A, *p* < 0.05. **(C)** Effect of transfection with AEG-1 siRNA on AEG-1 and TS protein (left panel) and gene expression (right panel) levels was detected using western blotting and RT-PCR. *: compare with PC-9, *p* < 0.05; **: compare with PC-9R-A, *p* < 0.05. **(D)** Flow cytometry dot plots and quantitative analysis showed increased apoptosis rate in PC-9R-A cells after AEG-1 knockdown. *p < 0.05.

To model therapeutic-induced chemoresistance *in vitro*, we generated a PC-9 cell-line (PC-9R-A) that exhibited resistance to pemetrexed, as confirmed by relative IC_50_ values obtained from the MTS assay (Figure 2A). We also found that expression levels of AEG-1 and TS mRNAs were higher in PC-9R-A cells than in parental PC-9 cells, as detected by quantitative RT-PCR (Figure 2B).

To determine whether TS expression positively regulated by AEG-1 was associated with the resistance to pemetrexed in NSCLC, we knocked down AEG-1 by transfecting PC-9R-A cells with AEG-1 siRNA. The results of western blotting and RT-PCR experiments revealed that knockdown of AEG-1 significantly decreased AEG-1 and TS mRNA and protein levels compared to those detected in cells treated with mock siRNA (Figure 2C).

To test whether downregulation of AEG-1 could reverse drug resistance, we analyzed pemetrexed efficacy in PC-9R-A cells, in which AEG-1 was knocked down by AEG-1 siRNA. We observed that AEG-1 knockdown was associated with lower values of pemetrexed relative IC_50_ than those noted in untreated PC-9R-A cells (Figure 2A and 2B). In addition, apoptosis rate was also higher after treatment with AEG-1 siRNA (Figure 2D).

These findings indicated that TS expression might be regulated by AEG-1 and that this relationship plays a role in the development of resistance to pemetrexed chemotherapy. AEG-1 knockdown affords a possibility of reversing the resistance to pemetrexed in NSCLC.

### AEG-1 overexpression induced TS expression and resistance to pemetrexed

**Figure 3:**
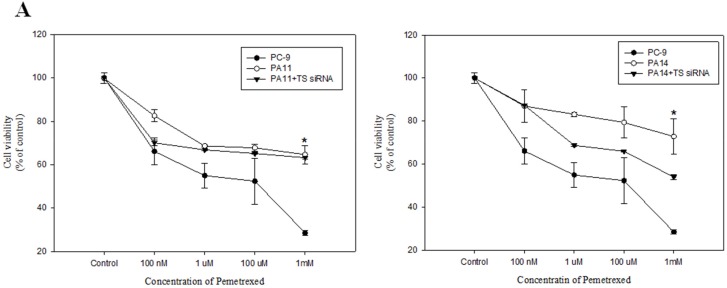

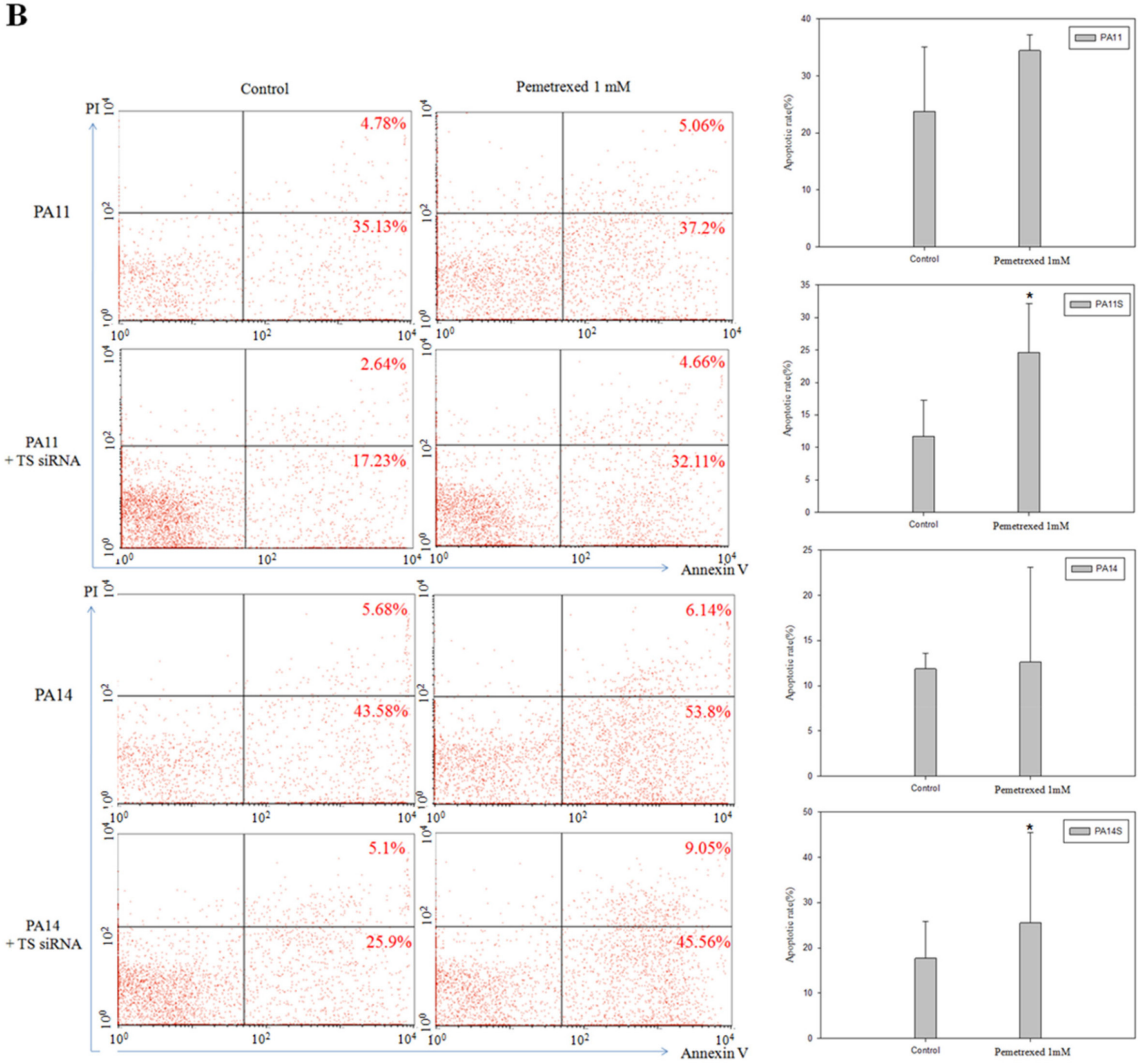

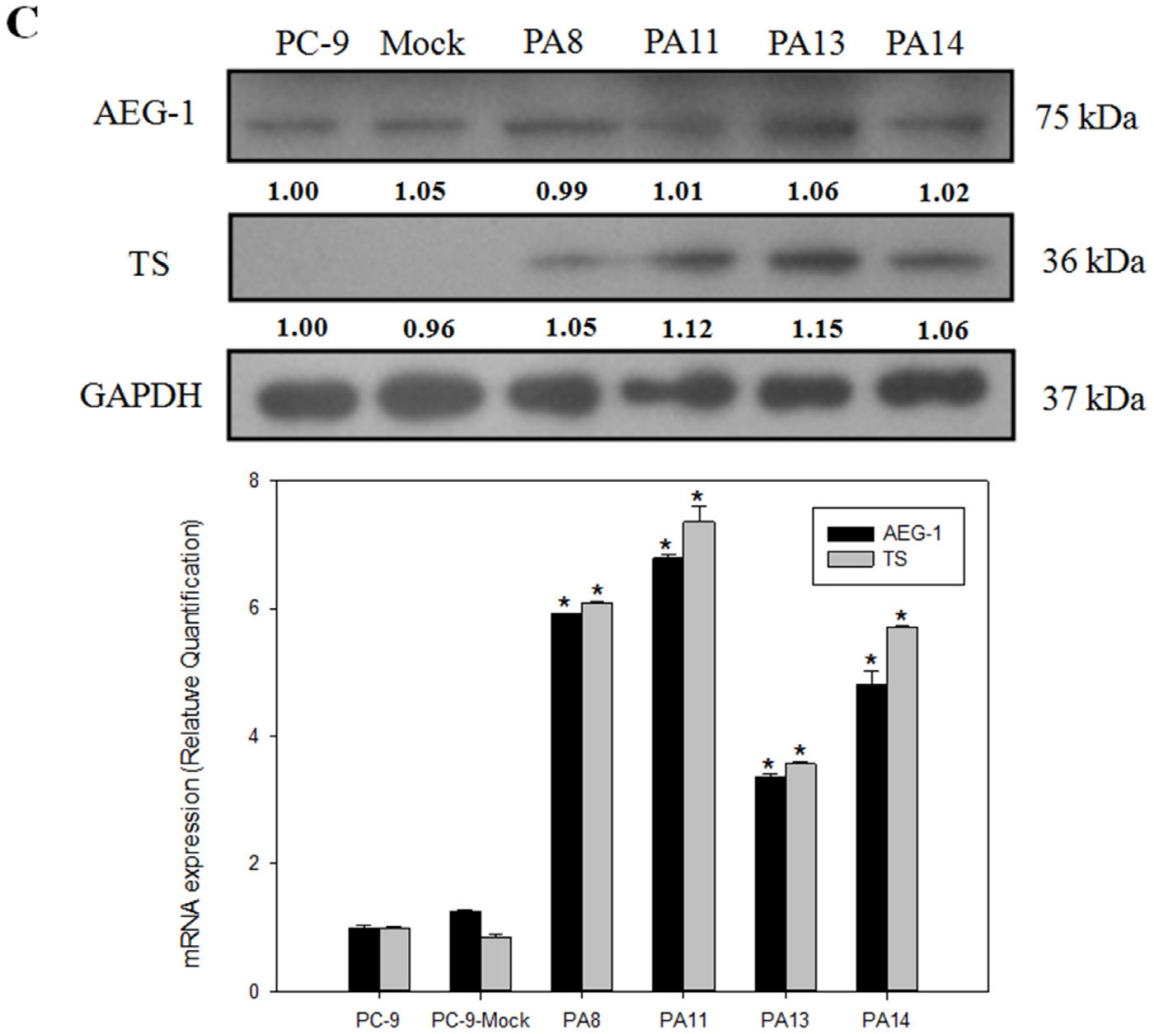

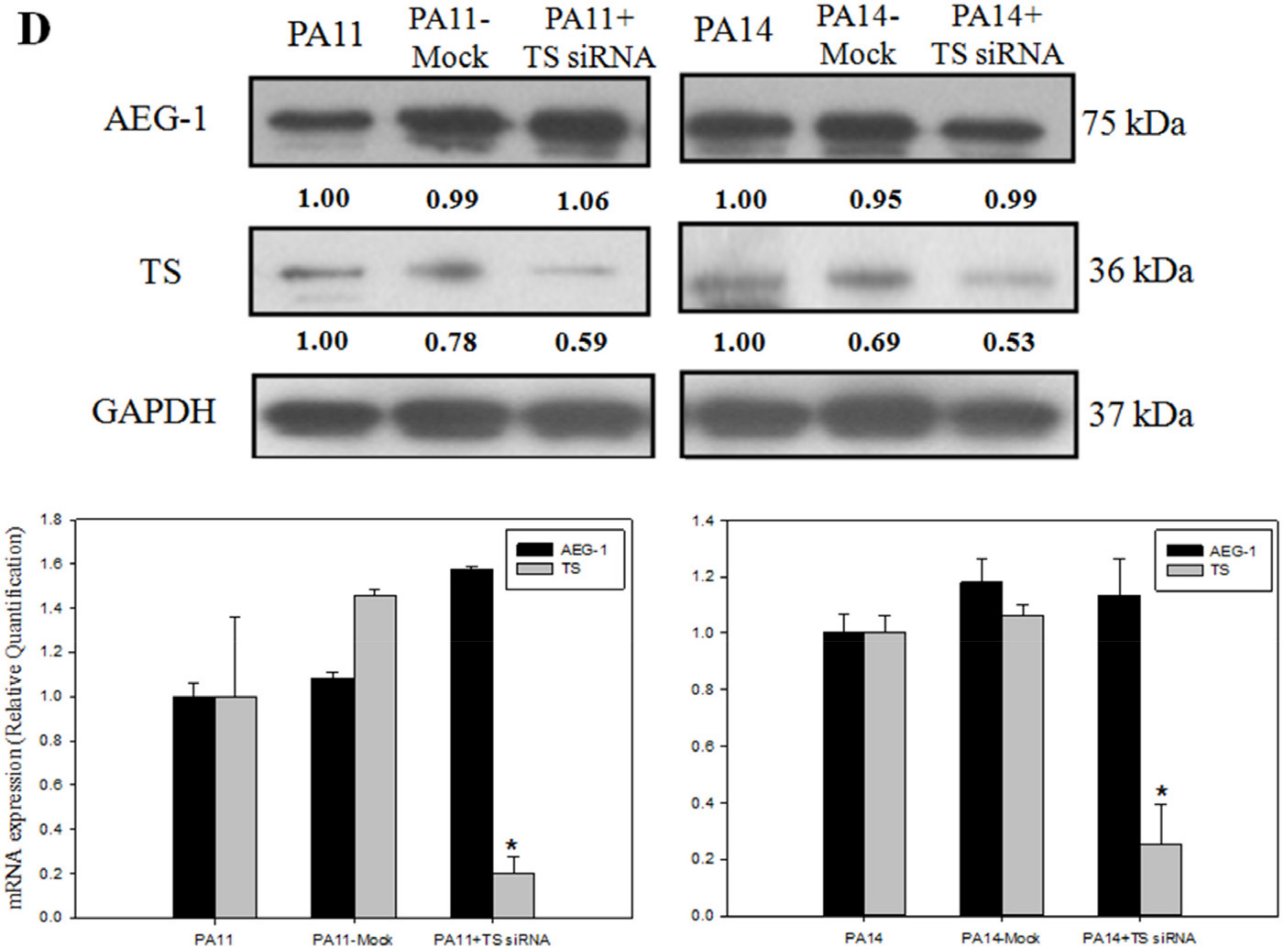
Overexpression of astrocyte-elevated gene-1 induced thymidylate synthase expression and elevated resistance to pemetrexed **(A)** MTS assays demonstrated that astrocyte-elevated gene-1 (AEG-1) overexpressing PA11 and PA14 cell lines were more resistant to pemetrexed action than the primary PC-9 cell line. *p < 0.05. Transfection of cells with thymidylate synthase (TS) siRNA led to partial restoration of pemetrexed inhibitory action on cell viability in AEG-1 overexpressing cell lines PA11 and PA14. **(B)** Flow cytometry dot plots and quantitative analysis showed increased apoptosis rate in PA11 and PA14 cell lines after TS knockdown. *p < 0.05. **(C)** Western blotting and RT-PCR demonstrated that overexpression of AEG-1 in cell lines PA8, PA11, PA13, and PA14 led to relatively higher levels of TS expression. *: compare with PC-9, *p* < 0.05. **(D)** Decreased TS protein and mRNA levels following transfection of AEG-1-overexpressing cell lines PA11 and PA14 with TS siRNA were detected with western blotting and RT-PCR, respectively. *: compare with PA11 and PA14, *p* < 0.05, respectively.

To further confirm the link between AEG-1 and pemetrexed resistance in lung cancer cells, we established an AEG-1-overexpressing lung cancer cell line. Overexpression of AEG-1 in all clones resulted in increased TS expression (Figure 3C). Furthermore, overexpression of AEG-1 in PC-9 cell line (clone PA11 and PA14) led to a higher value of pemetrexed IC_50_ (Figure 3A). This was consistent with the finding that PC-9R-A (pemetrexed-resistant cell line) had a higher expression of AEG-1 and TS when compared with the primary PC-9 cell line (Figure 2C).

To confirm that the positive relationship between AEG-1 and TS expression levels was associated with the resistance to pemetrexed in NSCLC, we performed TS knockdown by transfecting AEG-1-overexpressing cell lines PA11 and PA14 with TS siRNA. TS knockdown was associated with relative pemetrexed IC_50_ values that were higher than in untreated PC-9 cells, but lower than in AEG-1-overexpressing PA11 and PA14 clones (Figure 3A). In addition, TS knockdown increased apoptosis of AEG-1 overexpressing PA11 and PA14 cells (Figure 3B).

These findings indicated that TS expression can be upregulated by AEG-1 and that this positive relationship is essential for the development of resistance to pemetrexed chemotherapy.

### Expression levels of TS and excision repair cross-complementation group 1 (ERCC1) correlated with therapy response in advanced lung adenocarcinoma treated with pemetrexed plus platinum

From July 2012 to July 2014, in our hospitals, we admitted 115 patients with lung adenocarcinoma, which were treated with pemetrexed plus platinum as the first-line chemotherapy. Thirty-three patients were excluded because they had a sensitizing epidermal growth factor receptor mutation (exon 19 deletion and L858R) and therefore, received treatment with a tyrosine-kinase inhibitor before chemotherapy. For another five patients, chemotherapy response could not be evaluated. In the end, 77 patients completed chemotherapy treatment and agreed to provide clinical data for analysis.

The median age of patients was 61 years, and 42 of 77 patients were males (54.5%). The majority of the patients never smoked (*n* = 51, 66.2%); 60 patients (77.9%) used cisplatin as their first-line doublet chemotherapy, and most of them had stage IV disease at presentation (*n* = 58, 75.3%).

Because the expression of ERCC1 has been reported to be associated with platinum efficacy in NSCLC patients [31], we analyzed the relationship between expression levels of TS and ERCC1 and efficacy of pemetrexed plus platinum treatment. TS and ERCC1 expression levels were examined in tumor tissues of the 77 patients using immunohistochemical staining. The median TS H-score was 40 (range 0–270), and the median ERCC1 H-score of was 30 (range 0–270). These values were used as division lines to classify tumor tissues as those with high and low TS and ERCC1 expression levels. By using H-scores, we revealed that TS expression level positively correlated with that of ERCC1 (R = 0.692, R^2^ = 0.479, *P* < 0.001). There were no other significant associations between clinical characteristics, including age, gender, smoking status, clinical stages, and expressions levels of TS and ERCC1 (Table 1).

**Table 1:** Comparison of biological and clinical characteristics in groups of patients treated with pemetrexed plus platinum as first-line chemotherapy and stratified by expression levels of thymidylate synthase, excision repair cross-complementation group 1, or astrocyte-elevated gene-1 proteins

	Low TS vs. high TS *n* (%)	*P* value	Low ERCC1 vs. high ERCC1 *n* (%)	*P* value	Low AEG-1 vs. high AEG-1 *n* (%)	*P* value
**Age**						
< 60 (*n* = 37)	22 (59.5%) vs. 15 (40.5%)	0.293	20(54.1%) vs. 17 (45.9%)	0.722	22 (59.5%) vs. 15 (40.5%)	0.293
≥ 60 (*n* = 40)	19 (47.5%) vs. 21 (52.5%)		20 (50.0%) vs. 20 (50.0%)		19 (47.5%) vs. 21 (52.5%)	
**Gender**						
Male (*n* = 42)	26 (61.9%) vs. 16 (38.1%)	0.095	26 (61.9%) vs. 16 (38.1%)	0.055	24 (57.1%) vs. 18 (42.9%)	0.453
Female (*n* = 35)	15 (42.9%) vs. 20 (57.1%)		14 (40.0%) vs. 21 (60.0%)		17 (48.6%) vs. 18 (51.4%)	
**Smoking**						
Never (*n* = 26)	15 (57.7%) vs. 11 (42.3%)	0.577	14(53.8%) vs. 12 (46.2%)	0.812	14(53.8%) vs. 12 (46.2%)	0.940
Current or ever (*n*= 51)	26 (51.0%) vs. 25 (49.0%)		26 (51.0%) vs. 25 (49.0%)		27 (52.9%) vs. 24 (47.1%)	
**Stage**						
IIB (*n* = 19)	10 (52.6%) vs. 9 (47.4%)	0.951	12 (63.2%) vs. 7 (36.8%)	0.260	9 (47.4%) vs. 10 (52.6%)	0.554
IV (*n* = 58)	31 (53.4%) vs. 27 (46.6%)		28 (48.3%) vs. 30 (51.7%)		32 (55.2%) vs. 26 (44.8%)	
**Treatment response**						
PR (*n* = 38)	22 (57.9%) vs. 16 (42.1%)	0.04	22 (57.9%) vs. 16 (42.1%)	0.088	23 (60.5%) vs. 15 (39.5%)	0.206
SD or PD (*n* = 39)	14 (35.9%) vs. 25 (64.1%)		15 (38.4%) vs. 24 (61.5%)		18 (36.2%) vs. 21 (53.8%)	

AEG-1, astrocyte-elevated gene-1; ERCC1, excision repair cross-complementation group 1; PD, progressive disease; PR, partial response; SD, stable disease; TS, thymidylate synthase

Treatment responses were as follows: 38 patients (49.3%) showed a PR, 20 patients (26.0%) showed no change (stable disease, SD), and 19 patients (24.7%) experienced progressive disease (PD). Patients who had lower TS expression level tended to have better responses compared to those who were in the group with higher TS expression level (partial response [PR]: low TS vs. high TS, 57.9% vs. 42.1%; SD or PD: low TS vs. high TS, 35.9% vs. 64.1%, *P* = 0.04, respectively) (Table 1). Patients who had lower expression levels of both TS and ERCC1 had a relatively higher response rate (19/30, 63.3%) compared to those who presented with higher expression levels of these two proteins (14/35, 40.0%, *P* = 0.05).

### Expression of AEG-1 affected disease progression and survival

The median value of AEG-1 H-score was 120 (range 0–255) and, as in the case of TS and ERCC1, it was used to divide patients into those who had either high or low AEG-1 expression level in tumor tissues. Comparison of respective H-scores did not reveal a significant correlation between expression levels of TS and AEG-1 (R = 0.043, R^2^ = 0.002, *P* = 0.710).

**Figure 4:**
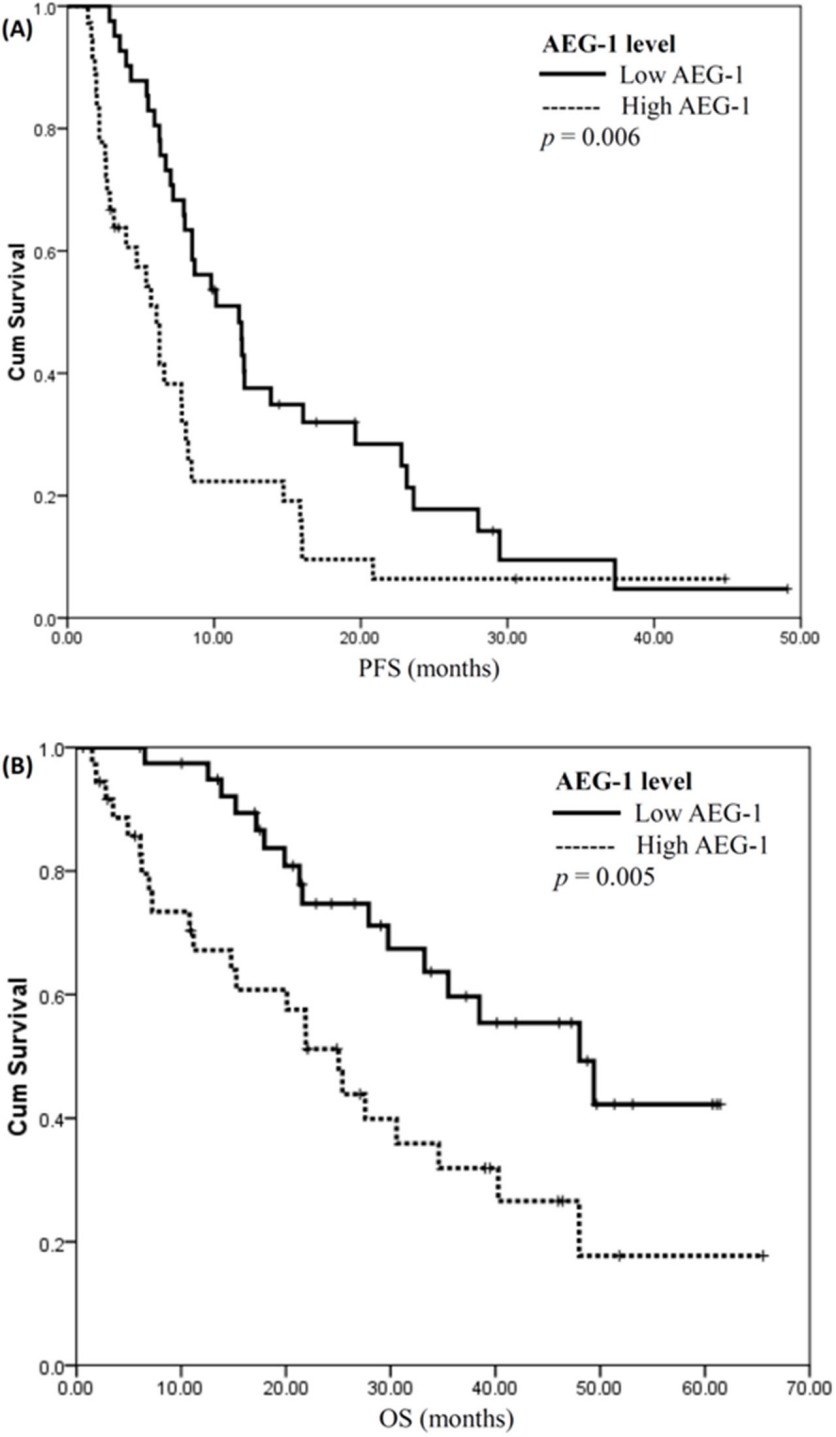
Expression level of astrocyte-elevated gene-1 affects disease progression and survival **(A)** Progression-free survival plots (PFS) for patients with low and high levels of astrocyte-elevated gene-1 (AEG-1) expression are illustrated. PFS for patients with low AEG-1 expression was significantly longer (low vs. high, 11.7 vs. 6.1 months; *P* = 0.006, log rank test). **(B)** Overall survival analysis (OS) for patients with low and high AEG-1 expression levels. OS for patients with low AEG-1 expression was significantly longer (low vs. high, 48.0 vs. 25.0 months; *P* = 0.005, log rank test).

Patients with low AEG-1 expression level had a significantly longer median progression-free survival (PFS) than those with high AEG-1 expression (11.7 vs. 6.1 months; *P* = 0.006) (Figure 4A). In addition, the difference in overall survival (OS) was also statistically significant between groups of patients with low and high AEG-1 expression levels (median: 48.0 vs. 25.0 months; *P* = 0.005) (Figure 4B). In contrast, there were no statistically significant differences in PFS and OS between groups of patients with low and high levels of TS or ERCC1.

These results indicated that AEG-1 expression level could be a prognostic factor for the outcome of patients with lung adenocarcinoma treated with platinum plus pemetrexed as their first-line chemotherapy.

### Increased expression of AEG-1 and TS after acquired pemetrexed resistance in lung adenocarcinoma

**Figure 5:**
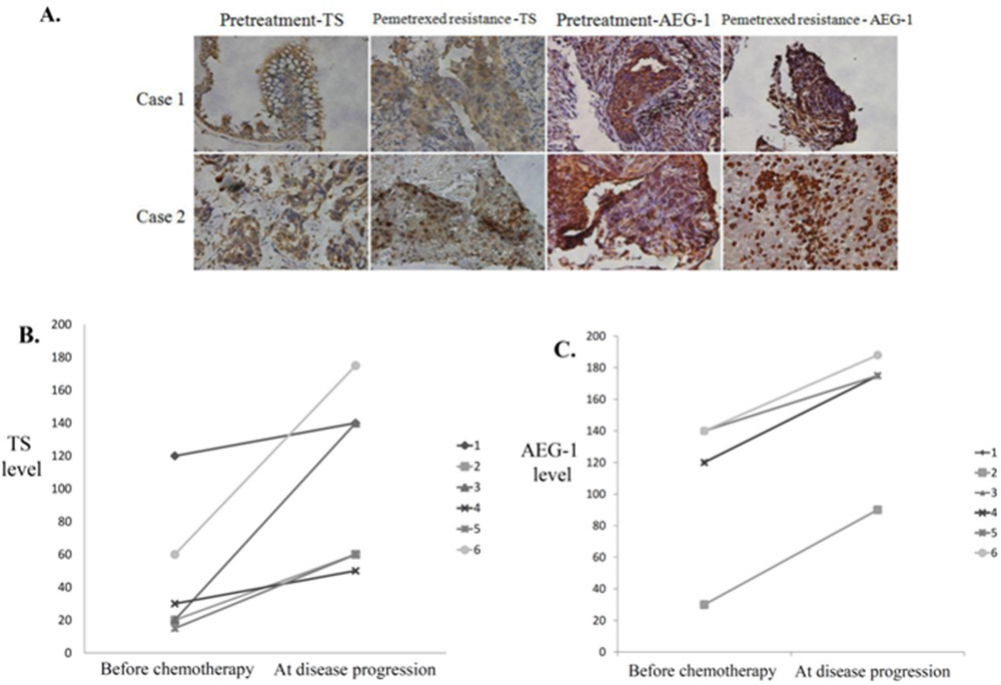
Increased expression of astrocyte-elevated gene-1 and thymidylate synthase after acquired pemetrexed resistance in lung adenocarcinoma **(A)** Immunohistochemical staining for thymidylate synthase (TS) and astrocyte-elevated gene-1 (AEG-1) proteins in tissue samples from two patients before pemetrexed treatment and after the development of pemetrexed resistance. **(B)** Immunohistochemical staining-derived H-scores for TS **(B)** and AEG-1 **(C)** expression levels were obtained in tissues from six patients before chemotherapy and at disease progression. Mean TS H-score increased from 44.2 at baseline to 104.2 at disease progression (*P* = 0.024), which was concomitant with a comparable increase of AEG-1 H-score from 115.0 to 163.0 (*P* < 0.001).

A total of six patients (three males and three females) had another biopsy after disease progression under first-line pemetrexed plus cisplatin followed by pemetrexed continuation maintenance therapy. In this cohort of patients, the initial reactions to first-line chemotherapy were five cases of PR and one case of SD. Immunohistochemical staining for AEG-1 and TS was performed in tissues of all these patients before chemotherapy and after disease progression (Figure 5A). The expression levels of TS and AEG-1 in post-progression tumors of each patient were higher than in pre-chemotherapy tumors. For TS, pre-chemotherapy H-score was in the range of 15–120, but it increased to 50–175 post-progression, whereas the mean TS H-score increased from 44.2 to 104.2, respectively (*P* = 0.024). For AEG-1, the pre-chemotherapy H-score range was 30–140, but it increased to 90–188 post-progression. Accordingly, mean pre-chemotherapy AEG-1 H-score was 115.0, and it increased to 163.0 post-progression (*P* < 0.001; Figure 5B and 5C).

These results suggested that increased expression of AEG-1 and TS in repeated biopsy samples contributed to the development of resistance to pemetrexed chemotherapy treatment and caused lung cancer progression in patients with lung adenocarcinoma.

## DISCUSSION

AEG-1 (also known as metadherin, MTDH, or lysine-rich CEACAM1 co-isolated, LYRIC) has emerged in recent years as a potentially crucial mediator of tumor malignancy and a key converging point of a complex network of oncogenic signaling pathways [32]. Here, we report that elevated expression of AEG-1 positively affects expression of TS and contributes to the development of resistance to pemetrexed chemotherapy in lung adenocarcinoma in ways that will need to be clarified in future studies. Our data indicate a significant correlation between TS and AEG-1 gene expression levels in NSCLC. Furthermore, we demonstrated that AEG-1 could potentially be a prognostic factor for NSCLC treatment outcome.

Many studies have shown that TS expression level could be suggestive of the objective response of patients with NSCLC treated with pemetrexed-containing chemotherapy [12–19]. ERCC1 is another predictive marker for platinum-based chemotherapy. TS and ERCC1 protein expression levels were associated with clinical outcomes in patients with pulmonary adenocarcinoma who were treated with pemetrexed plus platinum as their first-line chemotherapy [33, 34]. By using immunohistochemical staining, we evaluated TS and ERCC1 expression levels in biopsy specimens obtained from patients with pulmonary adenocarcinoma who had received pemetrexed plus platinum as their first-line treatment. We found that lower TS expression was significantly associated with a better response to treatment. A similar outcome was noted when both markers were combined: patients with lower levels of both TS and ERCC1 showed higher response rates. These results show that expression levels of TS and ERCC1 proteins can be predictive markers of responsiveness to chemotherapy in patients with pulmonary adenocarcinoma who were treated with pemetrexed plus platinum.

Although higher TS expression appeared to be associated with resistance to pemetrexed treatment [20, 21], the mechanism for enhanced TS expression and induction of resistance to chemotherapy in NSCLC has not been clarified. AEG-1 expression is an important hallmark of aggressive chemotherapy-resistant cancers. Previous studies have suggested that AEG-1 contributes to the mechanisms of resistance to a broad spectrum of various chemotherapeutics, including 5-FU, doxorubicin, paclitaxel, cisplatin, and 4-hydroxycyclophosphamide [26, 27, 35, 36]. In human hepatocellular carcinoma, AEG-1 augments the expression of transcription factors that regulate TS expression and induce resistance to 5-FU [27]. Our study is the first to reveal that increased expression of AEG-1 induces TS expression and contributes to the resistance of cancer cell lines to pemetrexed *in vitro*. Our conclusions about the negative correlation between AEG-1 and TS expression levels on one hand and sensitivity to pemetrexed on the other hand, drawn from experiments in cultured cells, were further confirmed in the analysis of repeated biopsy samples from patients with lung adenocarcinoma, which manifested with disease progression after pemetrexed treatment. Furthermore, we also showed that in cancer lines, overexpression of AEG-1 decreased sensitivity to pemetrexed, whereas downregulation of AEG-1 decreased the development of resistance to pemetrexed treatment. Because of the important contribution of AEG-1 to drug resistance, this protein could be a viable target of novel anticancer agents for a wide range of cancer types [36–38].

AEG-1 has a multifunctional role in tumor development as it is involved in multiple signaling pathways [32]. Regulation of activity of these signaling pathways by AEG-1 represents an additional way whereby AEG-1 may cause drug resistance in cancer [26, 28, 29]. Activation of the PI3K/AKT pathway and c-Myc also promote the development of resistance to lung cancer treatment [39]. Recent studies have revealed that TS expression is upregulated via the activation of the PI3K/AKT pathway [40] and c-Myc [41]. Our data suggest that AEG-1 induces resistance to chemotherapy via an increase in TS expression. Further investigations about signaling pathways involved in the elevation of TS expression by AEG-1 that results in the resistance to pemetrexed in NSCLC will be explored in our future studies.

Numerous studies have frequently observed high levels of AEG-1 expression in cancer patients, which correlated with poor clinical outcomes [22, 23]. High AEG-1 expression leads to disease progression in NSCLC [24, 25]. A recent retrospective study confirmed that AEG-1 expression level analyzed by immunohistochemistry was associated with clinical/pathological stage [42]. In multivariate analysis, AEG-1 expression was found to be an independent prognostic factor for both overall and disease-free survival after postoperative chemotherapy and radiotherapy [42]. Our study revealed that among lung adenocarcinoma patients who received pemetrexed plus platinum as their first-line chemotherapy, individuals with lower AEG-1 expression had a significantly longer median PFS and OS than those with higher AEG-1 expression. Thus, our data confirmed the notion that AEG-1 expression could be an independent prognostic factor for patient outcome. Our results not only suggest a potentially promising use of AEG-1 level as a valuable prognostic indicator, but also imply a possible link between biological functions of AEG-1 in cancer progression and induction of resistance to chemotherapy on one hand, and NSCLC pathogenesis on the other hand. This knowledge could lead to the development of a novel NSCLC treatment strategy.

Our findings demonstrate a significant positive correlation between TS and AEG-1 expression levels in NSCLC cell lines. However, according to the analysis of corresponding H-scores in biopsy samples from patients with lung cancer, TS expression did not significantly correlate with that of AEG-1. This apparent discrepancy may be caused by at least two reasons. First, lung cancer tumor components are heterogeneous, so immunohistochemical staining of cells obtained from small clinical biopsy samples may not have been sufficiently representative. Second, H-score evaluation is semiquantitative. It may be difficult to quantify protein expression by using visual scoring of immunohistochemical assay results, so other, more precise quantitative methods for assessment of TS and AEG-1 expression levels are needed. Until a proper validation of such methods, the discrepancy between observations of TS and AEG-1 levels in cell lines and in patient biopsy samples will remain the major limitation of conclusions drawn from this study.

## CONCLUSION

We have found that modulation of AEG-1 levels in lung cancer cell lines caused parallel changes in TS expression and sensitivity to pemetrexed. Furthermore, AEG-1 levels negatively correlated with patient survival and upregulation of AEG-1 and TS expression accompanied the progression of NSCLC. Therefore, it is likely that chemoresistance-promoting role of AEG-1 in NSCLC is mediated through the upregulation of TS. Understanding the roles of AEG-1 in NSCLC progression will not only advance our knowledge of the mechanisms underlying chemoresistance in NSCLC, but will also clarify if modulation of AEG-1 and associated signaling pathways is a viable therapeutic strategy for NSCLC treatment.

## MATERIALS AND METHODS

### Cell lines and culture conditions

A549, H157, H520, and H292 human lung cancer cell lines were obtained from the American Type Culture Collection Cell bank, whereas CL1-0, CL1-5, PC-9, and H1975 human lung cancer cell lines were obtained from the Institute of Biomedical Sciences, Academia Sinica. Cells were cultured in RPMI-1640 medium supplemented with 10% fetal bovine serum (FBS).

To generate a pemetrexed-resistant lung cancer cell line, PC-9 cells, a pemetrexed-sensitive cell line, were seeded at a low density and exposed to increasing concentrations of pemetrexed. After approximately six months of continuous pemetrexed exposure, we established a pemetrexed-resistant cell clone (PC-9R-A), which was maintained on pemetrexed with a concentration of 750 nM.

To produce an AEG-1 overexpressing cell line, PC-9 cells were transfected with the pcDNA3.1 empty vector or AEG-1-pcDNA 3.1 vector (also known as Metadherin-pcDNA 3.1; Life Technologies, Carlsbad, CA, USA) using Lipofectamine 2000 reagent (Invitrogen, Carlsbad, CA, USA). After 24 h, fresh RPMI-1640 containing 300 mg/mL zeocin was added. The culture medium was replaced three times weekly, until stably transfected colonies were observed.

### *In vitro* MTS assay

Cells were cultured at a density of 5,000 per well in 96-well culture plates. To assess cell viability, stepwise 10-fold dilutions of the anticancer drug were added 2 h after plating, and the cultures were incubated at 37 °C for 72 h. At the end of the culture period, 20 μL of MTS solution (CellTiter 96 Aqueous One Solution Cell Proliferation Assay; Promega, Madison, WI, USA) was added, and the cells were incubated for another 4 h. Then, the absorbance was measured at 490 nm using an enzyme-linked immunosorbent assay plate reader. Chemosensitivity was expressed in terms of values of drug concentrations producing 50% growth inhibition.

### Plasmids, siRNAs, and transient transfection

All plasmids and siRNAs were obtained from Life Technologies (Carlsbad, CA, USA). Transfection was carried out using Lipofectamine 2000 (Invitrogen) according to the manufacturer’s instructions. Before transfection, cells were grown in 24-well plates with RPMI-1640 medium in the absence of antibiotics or FBS. To knock down AEG-1, cells were cultured for 48 h after transfection with 100 nM siRNA for AEG-1 using Lipofectamine 2000. Downregulation of AEG-1 expression was then measured by western blotting and real-time PCR.

### Western blot analyses

Cell lysates were fractionated by using sodium dodecyl sulfate-polyacrylamide gel electrophoresis for western blot detection of respective proteins. The primary antibodies used were anti-AEG-1 (1:1000; rabbit polyclonal; Invitrogen), anti-TS (1:1000; mouse monoclonal; Abcam), and anti-GAPDH (1:10000; mouse monoclonal; St John’s Laboratory).

### RNA extraction and quantitative real-time PCR

Total RNA was extracted using a Qiagen mRNA easy mini kit (Qiagen). First-strand complementary DNA synthesis was performed from 2 μg of total RNA by using Invitrogen SuperScript Reverse Transcriptase (Thermo Fisher Scientific, Carlsbad, CA, USA). Real-time PCR was performed according to the manufacturer’s protocol for Applied Biosystems Power SYBR® Green PCR Master Mix, and relative AEG-1 and TS mRNA expression levels were determined as a ratio to that of actin mRNA.

### Annexin V-FITC flow cytometry analysis

Movement of phosphatidylserine to the outer membrane upon apoptotic induction was detected by Annexin V-FITC staining (BD Biosciences, USA). Lung cancer cell lines were treated with pemetrexed (0 and 1 mM) for 72 h and then, the cells were harvested, incubated with Annexin V-FITC and propidium iodide for 15 min at room temperature, and analyzed by using BD FACS Calbur™ flow cytometry. A minimum of 5,000 cells were acquired per sample and represented in dot plot.

### Patients and data collection

This study was approved by the investigational review boards of the National Taiwan University Hospital, and all participating patients provided informed consent. Patients with a diagnosis of locally advanced or metastatic nonsquamous cell lung cancer, stage IIIB or IV, and those who were treated with pemetrexed plus platinum as first-line chemotherapy regimen in the National Taiwan University Hospital and National Taiwan University Hospital Yunlin Branch were potentially eligible for inclusion. The diagnosis of NSCLC was based on histological examination of biopsy specimens or on cytological analysis of samples aspirated with a fine needle. Patients’ basic data and medication usage were retrieved from electronic medical records.

The patients were administered with cisplatin or carboplatin plus pemetrexed intravenously every three weeks. Furthermore, patients received 500 mg/m^2^ pemetrexed intravenously every three weeks as continuation maintenance therapy if the disease did not progress after 4–6 cycles of the first-line treatment. All patients were adults with good performance status (Eastern Cooperative Oncology Group Performance Status: 0–1). The medical records were analyzed for age, gender, tumor staging, histology type, treatment response, and survival.

Treatment was discontinued if there was evidence of progressive disease, or unacceptable toxicity, or if the patient decided so. Evaluation of the best tumor response rate was performed at the end of the treatment period by the Response Evaluation Criteria in Solid Tumors (RECIST version 1.1) [30] and defined as partial response (PR), stable disease (SD), or progressive disease (PD). Acquisition of tumor tissue by another biopsy was performed at the onset of disease progression after chemotherapy, if deemed feasible.

Progression-free survival (PFS) was defined as the time from the first cycle of pemetrexed plus platinum to documented progression or death from any cause, and was censored at the date of the last follow-up visit for patients who were still alive and who had not exhibited progression. The overall survival (OS) was measured from the date of the start of the treatment to the date of the last follow-up. Patients were followed up until August 2015.

### Immunohistochemical analysis

Lung cancer tumor specimens were examined for the expression of TS, excision repair cross-complementation group 1 (ERCC1), and AEG-1 by immunohistochemical approaches. Antibodies against TS (1:200, Abcam, ab58287), ERCC1 (1:200, Thermo, MS-671-P), and AEG-1 (1:200, Invitrogen, 406400) were left to react at 4 °C overnight. By using breast carcinoma tissue with high AEG-1 expression as a positive control, tonsil tissue for TS positive control, and lung carcinoma tissue for ERCC1 positive control, expression levels of TS, ERCC1, and AEG-1 for each tumor were comparatively assessed. Tumors were considered to be positive, when cell cytoplasm was stained. Staining density was evaluated and classified as no staining, weak, intermediate, or strong staining. The percentages of positive neoplastic cells were also determined.

Overall expression levels of TS, ERCC1, and AEG-1 were analyzed using a semiquantitative histologic score (H-score) defined as the degree of tumor cell staining density (0, none; 1, weak; 2, intermediate; 3, strong) multiplied by the percentage of positive neoplastic cells.

### Statistical analysis

H-scores for TS, ERCC1, and AEG-1 signals were reported in medians and ranges. The relationship of these H-scores was estimated by linear regression. The patients were divided into two groups by using the median H-scores for TS, ERCC1, and AEG-1 as group division lines. Correlation of H-scores with patients' clinical characteristics was estimated by using the Pearson’s χ^2^ test.

Categorical measures were expressed as percentages of subjects that manifested respective characteristics relative to the total number of subjects in the group. Quantitative variables were compared using independent Student’s *t*-tests or paired *t*-tests, if the samples from the same patient were repeatedly analyzed. Kaplan-Meier survival curves (K-M curves) were used to represent PFS and OS. Statistical calculations were performed using SPSS (Statistical Package for the Social Sciences), version 16.0 for Windows (SPSS Inc.). Differences were considered significant if *P* value was less than 0.05.
